# Nanoindentation Characterization of a Ternary Clay-Based Composite Used in Ancient Chinese Construction

**DOI:** 10.3390/ma9110866

**Published:** 2016-10-26

**Authors:** Dongwei Hou, Guoping Zhang, Rohit Raj Pant, Jack S. Shen, Mingming Liu, Hao Luo

**Affiliations:** 1 State Key Laboratory of Ocean Engineering, Department of Civil Engineering, Shanghai Jiao Tong University, Shanghai 200240, China; houdw@sjtu.edu.cn (D.H.); slshen@sjtu.edu.cn (J.S.S.); 2 Department of Civil & Environmental Engineering, University of Massachusetts Amherst, Amherst, MA 01003, USA; 3 Department of Civil and Environmental Engineering, Louisiana State University, Baton Rouge, LA 70803, USA; rpant1@lsu.edu; 4 College of Ocean Environment and Engineering, Shanghai Maritime University, Shanghai 200135, China; 7499870@gmail.com (M.L.); lh15801738182@gmail.com (H.L.)

**Keywords:** ternary clay-based composite, nanoindentation, C-S-H, geopolymer

## Abstract

Ternary clay-based composite material (TCC), composed of lime, clay and sand, and usually modified with sticky rice and other organic compounds as additives, was widely used historically in Chinese construction and buildings due to its high mechanical performance. In this study, to gain an insight into the micromechanical mechanism of this cementitious material, the nanomechanical properties and volume fraction of mechanically different phases of the binder matrix are derived from the analysis of grid nanoindentation tests. Results show that there are five distinct mechanical phases, where the calcium silicate hydrate (C-S-H) and geopolymer present in the binder matrix are almost identical to those produced in ordinary Portland cement (OPC) and alkali-activated fly-ash geopolymer materials in nano-mechanical performance. The nano-mechanical behavior of calcite produced by the carbonation of lime in this binder is close to the calcite porous outer part of some sea urchin shells. Compared to OPC, the C-S-H contained in the TCC has a relatively lower ratio of indentation modulus to indentation hardness, implying a relatively lower resistance to material fracture. However, the geopolymer and calcite, at nearly the same volume content as the C-S-H, help to enhance the strength and durability of the TCC by their higher energy resistance capacity or higher strength compared to the C-S-H. Rediscovering of TCC offers a potential way to improve modern concrete’s strength and durability through synergy of multi-binders and the addition of organic materials if TCC can be advanced in terms of its workability and hardening rate.

## 1. Introduction

Significant carbon emissions and high energy consumption in the production of Portland cement, as well as the durability problems occurring in modern concrete materials and structures, have prompted increasing interest in the search for greener and more sustainable cementitious materials for construction. A possible solution for these challenges may be found in the use of historical building materials, which tended to be more environmentally friendly, durable, and sustainable. Historically, lime-based mortar was widely used in ancient Greece [1,2,3,4,5,6], and pozzolan-based hydraulic materials [3,6] were extensively used in Europe and Western Asia until the invention of modern Portland cement in the 19th century in the UK. In ancient China, lime was also produced for construction as a building material about 5000 years ago [6,7]. Perhaps because of the absence of natural materials, such as volcanic ash, hydraulic mortar technology was not developed in ancient China [6]. However, a special hybrid ternary clay-based composite (TCC) composed of lime, clay, and sand was invented in the late Yangshao period (5000–3000 BC) [6]. It, subsequently, had widespread applications in various structures, for example, royal palace buildings, fortress walls, residential houses, water conservation facilities, and tombs. At least since the early Northern and Southern Dynasty (386–581 AD), organic constituents such as sticky rice soup, vegetable juice, egg white, brown cane sugar solution, tung oil, fish oil, and animal blood, have been introduced to improve the performance of the TCC materials significantly [6,8,9,10,11]. It may be the first widespread inorganic-organic composite mortar technology in China, or even in the world. Many surviving ancient Chinese structures, such as the Qiantang River Embankment, the Ming Dynasty City Wall, and the Hakka House, are still in service after many centuries, demonstrating TCC’s excellent durability.

TCC was usually synthesized from the three major constituents at different mix ratios for different purposes or applications for a particular type of structure. According to a Chinese encyclopedia published in 1637 during the Ming Dynasty (1368–1644 AD) [7], “The Exploitation of the Works of Nature”, TCC was first produced by mixing clay and lime at a mass ratio of 2:1, blending the mixture with river sand as aggregate or filler, and then adding sticky rice soup or carambola vine juice as the organic ingredient to make a moist mixture, which was then rammed and tamped to form different structural shapes. However, if the mixture was compacted in a relatively dry state, a more appropriate mix ratio of clay:lime:sand (by mass) could be 4:3:3 or 5:3:2 [12]. The TCC materials found in surviving ancient buildings are still “as hard as stone” and can be used to “sharpen a knife and axe” [6].

From the perspective of modern cement material science, the high strength of the ancient cementing material is attributed to the carbonation of lime (calcium carbonate) and the reaction between lime and clay (composed of amorphous silicates) to form both the aerial phases and hydraulic phases with the addition of water [5]. The addition of sticky rice can improve the binder material in both mechanical and penetration performance by restraining the formation of crystalline calcium carbonate and integrating with the calcium carbonate particles at a nanoscale level [6,10]. In essence, the TCC distinguishes itself from the lime-based mortar and the lime pozzolanic mortar used in ancient Europe, owing to an additional important and functional constituent, starch—a natural organic biopolymer, although they may function for the same purposes or have similar performance. Meanwhile, because of the inclusion of micron-sized aluminosilicate clay minerals, it is also remarkably different from the binary mortar, a lime-starch mixture binder also invented and used in ancient China [6].

In this paper, to understand the mechanical behavior of the TCC material at the microscale, nanoindentation tests were performed on TCC samples. Nanoindentation is a widely used technique to assess the elastic modulus and hardness of the bulk homogenous material. Recently, developments in this technique, such as the continuous stiffness measurement (CSM) and grid nanoindentation, have extended to the micro-mechanical characterization for heterogeneous materials, such as composites, polymers, biomaterials, and geomaterials [13,14]. In this paper, the grid nanomechanical properties and volume fraction of mechanically different phases of the ancient binding materials are derived from an analysis of nanoindentation tests. The main binder phases, calcite, C-S-H, and geopolymer products were distinct, and in these the C-S-H and geopolymer present in the TCC binder matrix were found to be almost identical to those produced in ordinary Portland cement (OPC) and alkali-activated fly-ash-based geopolymers, respectively, in nanomechanical performance, while the calcite produced by the carbonation of lime in TCC is close to the porous outer part of calcite in some sea urchin shells.

## 2. Materials and Methods

### 2.1. TCC Samples

**Figure 1:**
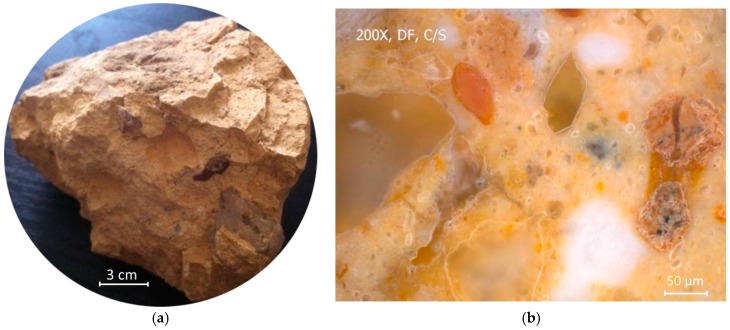
Original TCC sample (**a**) and its optical microscope image (**b**).

A ternary clay-based composite sample which is considerably tough, strong, and reportedly blast resistant, was obtained from an approximately 250 year old tomb in Anhui province in China. The sample seemed remarkably sound and showed no sign of deterioration, and had a significant amount of coarse-grained sand and gravel fractions within in the binder matrix, similar to the topology of modern concrete materials (shown in Figure 1).

### 2.2. X-ray Diffraction

Mineralogical analysis was first carried out to determine the mineral components of the TCC material using an X-ray diffraction method. A powdered sample was prepared from the scrapings of the freshly exposed TCC surface, excluding visually-observable coarse particles from the sample blocks, and this was then ground with anhydrous ethanol (used as coolant and lubricant) in a McCrone micronizing mill (Verder Scientific Inc., Nwetown, PA, USA) for three minutes to obtain binder particles smaller than 45 μm in size.

X-ray diffraction (XRD) was conducted on the powdered sample, which had a random particle orientation, in a Siemens D-5000 diffractometer (Karlsruhe, Germany) using Cu-K*α* radiation generated at 40 kV and 30 mA, using a scan range of 2°–70° (2*θ*, where *θ* is the diffraction angle). The scan used a 0.996° divergence slit, 0.501° scatter slit, and 0.1 mm receiving slit at a rotating speed of 1° 2*θ*/min and a step size of 0.02° 2*θ*. XRD patterns were analyzed both qualitatively and quantitatively. A computer program, Jade 9.0 (MDI, Livermore, CA, USA), was used to identify and match the XRD reflections, while semi-quantitative analysis was based on the methodology developed by Cook et al. [15], and an in-house computer program, XRDPhil (Philips electronic Co., Eindhoven, The Netherlands), was used to estimate the mass fractions of the major identified mineral phases.

### 2.3. Nanoindentation Testing

In preparing the samples for the indentation test, to obtain smooth surfaces for indentation, small chips 10–15 mm in size were first carefully cut and immersed in Epon 812 resin (Aremco Products Inc., Valley Cottage, NY, USA). After the resin hardened, the specimens were ground and polished with 400, 600, and 800 grit silicon carbide paper, followed by finer polishing using a low-nap polishing cloth and an alcohol-based diamond suspension with particle sizes of 3 μm and 1 μm. Surface roughness was measured using the optical profilometer and found to be of the order of 100–200 nm. Relatively smooth areas within the matrix were chosen for nanoindentation tests. Three grid indentation series were performed on two prepared samples. Trials ANC-1 and ANC-2 were performed on two separate locations in the same TCC specimen, while trial ANC-3 was performed on another sample.

**Figure 2:**
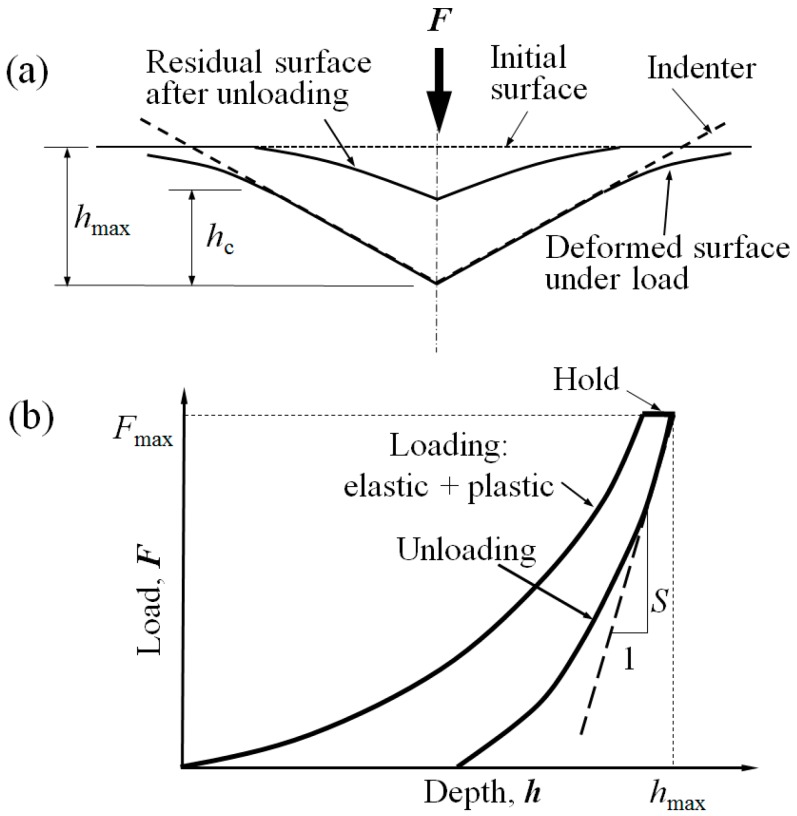
Schematic illustration of (**a**) nanoindentation loading and unloading processes and (**b**) the corresponding load–displacement curve.

Nanoindentation experiments were performed with an MTS Nano XP indenter (MTS Nano Instruments, Inc., Oak Ridge, TN, USA), which is a diamond Berkovich indenter with a tip radius of <20 nm, under the load control mode at room temperature. A trapezoidal loading profile was used for all tests, which consisted of five steps, as follows (Figure 2): (1) increase the load at a constant indentation strain rate of 0.05 s^−1^ to a pre-selected maximum indentation depth (*h*_max_) of about 200 nm; (2) hold the maximum load *F*_max_ constant for a given hold time *t*_h_ = 10 s, where *F* is the indentation load; (3) decrease *F* under the load control mode using the same loading rate (*dF*/*dt*) as that at *F*_max_ in the loading process, to 10% of *F*_max_; (4) hold the load (at 10% of *F*_max_) constant for 100 s to record the thermal drift of the instrument; and (5) decrease *F* linearly to zero.

Two mechanical properties, indentation modulus *E_r_*, and indentation hardness *H*, can be derived directly from the *F*-*h* curve [16,17]:

(1) $$E_{r}=\frac{\sqrt{\pi }}{2\beta \sqrt{A_{C}}}S$$

(2) $$H=\frac{F}{A_{C}}$$

(3) $$S=\frac{dF}{dh}{|}_{h_{\max}}$$

where *β* is a dimensionless correction factor for the indenter tip shape and *β* = 1.05 for the Berkovich indenter [17]. *S* is the unloading indentation stiffness and *A*_c_ is the contact area at *h*_max_, and is a polynomial function of the contact depth *h*_c_ at *h*_max_, (i.e., *A*_c_ = *f* (*h*_c_)) [16,17,18]. 

For isotropic homogeneous materials, the reduced modulus (*E*_r_) and the elastic modulus are related by the equation:

(4) $$\frac{1}{E_{r}}=\frac{\left(1-v^{2}\right)}{E}+\frac{\left(1-v_{i}^{2}\right)}{E_{i}}$$

where *E* and *v* are the elastic modulus and Poisson’s ratio of the tested material, respectively, and *E_i_* and *v_i_* are those of the indenter. In this paper, Poisson’s ratio *v* was assumed to be 0.2 for all measurements.

For a type of heterogeneous material composed of *N* different phases in a characteristic length scale *l*, indentation gives the bulk properties of material at length scale *L* ≈ 4 × *h*_max_ [19]. In the situation where *L* << *l*, each indentation event is a response of one of the individual phases in the material. Thus, if a large number of indentations are performed in a grid with spacing larger than the characteristic size of an individual phase, the probability of finding each phase is equal to the surface fraction occupied by this phase on the indentation surface. On the other hand, for *L* >> *l*, indentation gives an average response of the composite material. For a large number of indentations in arrays, statistical deconvolution of indentation results can be obtained by assuming the distribution of mechanical properties of each phase to be a Gaussian distribution [13,19,20]. Then, the cumulative distribution function (CDF) of each phase is given by:

(5) $$D(X_{i};\mu_{j}^{X},\sigma_{j}^{X})=\frac{1}{\sigma_{j}^{X}\sqrt{2\pi }}{\int_{-\infty }^{X_{i}}e^{-\frac{1}{2}\left(\frac{u-u_{j}^{X}}{\sigma_{j}^{X}}\right)}}^{2}du\;X=E,\;H$$

where, $\mu_{j}=\frac{1}{N_{j}}\sum_{i=1}^{N_{j}}X_{k}$ is the mean and $\sigma_{j}^{2}(x)=\frac{1}{N_{j-1}}\sum_{k=1}^{N_{j}}\left(x_{k}-\mu_{k}\right)$ is the standard deviation. Additionally, the surface fraction occupied by each phase, *f_j_*, is given by *f_j_* = *N_j_*/*N* subject to $\sum_{j=1}^{n}f_{j}=1$. The unknowns $\left\{f_{j},\mu_{j}^{M},\sigma_{j}^{M},\mu_{j}^{H},\sigma_{j}^{H}\right\}$ for *j* = 1, *n* were determined by minimizing the scaled experimental CDF and scaled model CDF [13]:

(6) $$\min\sum_{i=1}^{N}\sum_{X=M,H}{\left(\sum_{j=1}^{n}f_{j}D(X_{i};\mu_{j}^{X},\sigma_{j}^{X})-D_{X}(X_{i})\right)}^{2}$$

where $D_{X}(X_{i})=\frac{i}{N}-\frac{1}{2N}$ for *i* ∈ [1, *N*] gives the points of the experimental CDF. To avoid overlap of the two distributions, a further constraint was applied as: 

(7) $$\mu_{j}^{X}+\sigma_{j}^{X}\leq \mu_{j+1}^{X}+\sigma_{j+1}^{X}\;X=E,H$$

## 3. Results and Discussion

### 3.1. Mineral Components

**Figure 3:**
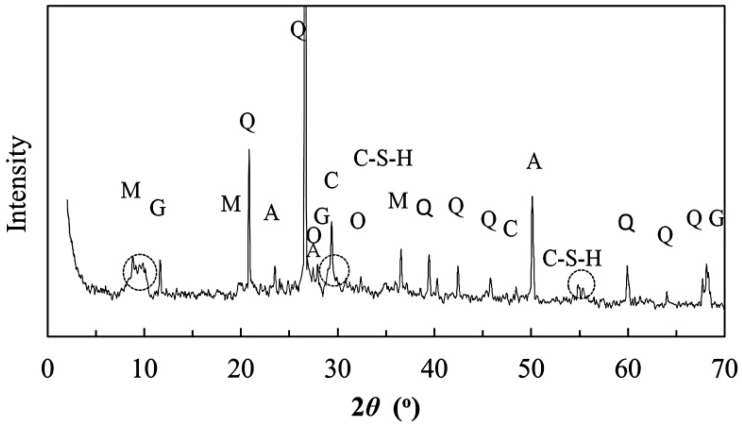
The XRD pattern of the powder matrix sample. (*Q*: Quartz, *M*: Muscovite, *O*: Orthoclase, *A*: Albite, *G*: Gypsum, and *C*: Calcite).

Figure 3 shows the XRD pattern of the fine-grained binder matrix. It is characterized by a series of strong and sharp reflections that are from highly crystalline minerals, including muscovite, gypsum, quartz, albite (a plagioclase feldspar), calcite, and orthoclase (a K-feldspar). The presence of a broad hump from ~20° to ~40° 2*θ* is characteristic of disordered, poorly-crystalline CSH and geopolymers [21]. It is well known that slow pozzolanic reactions take place between the alkaline hydrated lime (Ca(OH)_2_) and hydrous aluminosilicates (i.e., clay minerals) at ambient temperatures, leading to the formation of insoluble CSH, CAH (hydrated calcium aluminate), and CASH (hydrated calcium aluminosilicate). Meanwhile, nuclear magnetic resonance (NMR) spectra suggested the presence of three-dimensional aluminosilicate networks, which are characteristic of geopolymer products [22]. Joseph and Davidovits also found the amorphous phases composed of aluminosilicates and a zeolite like material (Na_2_O·Al_2_O_3_·4SiO_2_·2H_2_O) in ancient lime-pozzalantic materials [23]. Coexistence of C-S-H and geopolymer gels in the alkali activation of various aluminosilicate sources (metakaolin, fly ash) in the presence of calcium hydroxide was also demonstrated by Alonso and Palomo [24], Granizo et al. [25], and Yip et al. [26]. Owing to the dynamic nature of the dissolution and polymerization, several phases—the C-S-H from activation of silicate in clay, geopolymer product from the alkali activation of silicate and aluminosilication in clay, and the CaCO_3_ from the carbonation of lime—may coexist in the final product of TCC depending upon the hydrothermal condition (significant amounts of heat from the hydrolyzation of lime) and the high concentration of lime [24]. In the XRD pattern, the strong reflections from quartz actually mask the generally weak, broad reflections from clay minerals and other poorly-crystalline phases. Furthermore, the broadening of the peak corresponding to muscovite (8.8° 2*θ*) is indicative of the activation of the crystalline phase into a nano-crystalline or amorphous structure.

The results of a semi-quantitative analysis of the TCC binder are shown in Table 1. Apart from calcite and gypsum, the other crystalline phases mainly occur in the inert sand or gravel fraction. Calcite is the carbonation product of lime with atmospheric CO_2_, and gypsum may be present in the original raw material. All fine-grained phases with relatively weak or poor reflections are treated together, accounting for 37.0% of the hybrid binder. In the XRD pattern, the organic phase, the starch contained in the sticky rice, cannot be detected, which is most likely due to its amorphous state and small fraction.

### 3.2 Validation of Indentation Depth

The choice of indentation depth in a grid indentation technique is primarily governed by the length scale of the largest heterogeneity, *d*, and the microstructure, *D*. Based on microstructural analysis, the characteristic size of the fully-activated gel is of the order of 5–10 nm. The capillary pores in the C-S-H phase are also of a similar dimension and randomly distributed throughout [27,28]. Therefore, it is imperative that the indentation response of the C-S-H phase is inclusive of the effect of nano-porosity. In comparison, the microstructural length scale ranges from 1 μm to 4 μm and can be detected by SEM and TEM [29,30]. As unreacted clay, sand, or silt inclusions are crystalline, *d* is of the order of the lattice parameters. Based on SEM and TEM micrographs, *D* values for these phases are estimated to be between 1 μm and several micrometers. Therefore, indentation depth, *h* ∈ [200, 300] nm, is sufficient to be distinguishable and to satisfy the 1/10 rule for nanoindentation sensing of individual phase properties. However, this depth does not necessarily satisfy the roughness criteria, *R*_q_ < 3*h* [31], but efforts to bring *R*_q_ to below the 200 nm range without disturbing the sample surface presented challenges. In fact, in their nanoindentation study of shales, Bobko and Ulm [32] performed a similar experiment at this depth range with satisfactory results. Thus, this study concurs that the criteria for surface roughness can be relaxed, especially for a grid indentation technique that involves the statistical analysis of a massive volume of indentation data over a large area.

**Figure 4:**
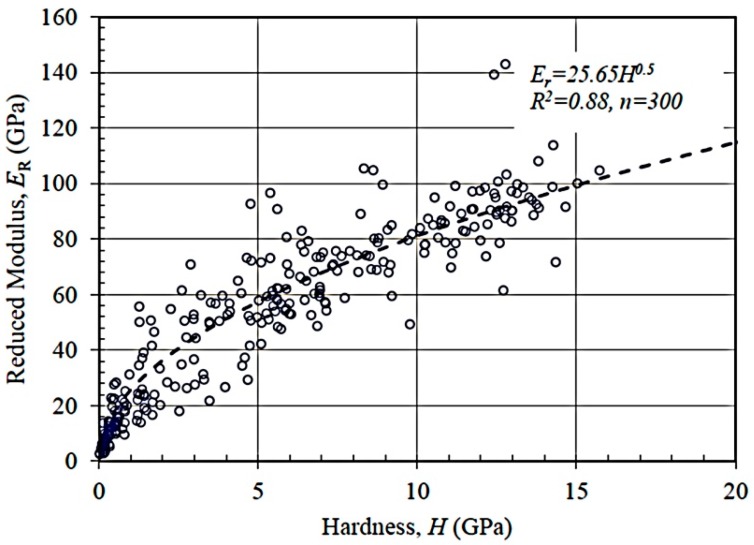
Relationship between indentation modulus and indentation hardness for TCC specimen ANC-1 (*h* = 200–210 nm, *n =* 300).

Figure 4 shows the plot of indentation modulus versus hardness for trial ANC-1. A good scaling relationship $E_{r}\propto \sqrt{H}$ confirms the relationship given by Equations (1) and (2), thereby implying the separation of scales in each indentation test and random sampling of the data. This also implies that the selected indentation depth has little effect on the validation of test results.

### 3.3. Statistical Analysis on Indentation Modulus and Hardness

**Figure 5:**
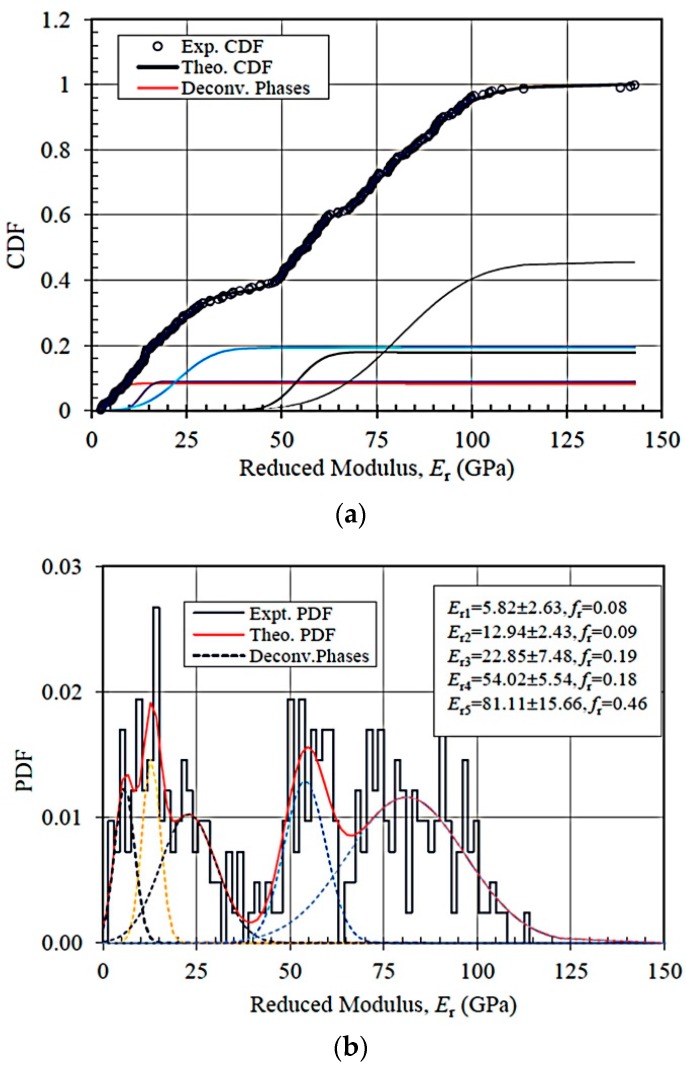
Reduced modulus deconvolution of ANC-1 data (**a**) CDF (**b**) PDF; *E*_ri_: reduced modulus of phase *i* (*i* = 1: microporosity; *i* = 2: mixed geopolymer-clay matrix; *i* = 3: C-S-H; *i* = 4: nano-crystallites; *i* = 5: inclusions); *f*_r_: volume fraction.

Figure 5 shows the deconvoluted peaks of the indentation modulus *E*_r_ for trial ANC-1 (*n* = 300) as an example, both in terms of the cumulative distribution function (CDF) (Figure 5a) and probability distribution function (PDF) (Figure 5b). Deconvolution of the indentation modulus CDF suggests five distinct mechanical phases present in the TCC material. The lowest reduced modulus of 5.82 ± 2.63 GPa and 12.94 ± 2.43 GPa are attributed to the pure clay inclusion with porous microstructures and mixed geopolymer-clay matrix, respectively. Similarly, *E*_r_ = 22.85~26.10 GPa indicates the indentation on the C-S-H phase. Peaks corresponding to *E*_r_ = 52.46~54.02 GPa result from the indentation on nano-crystallites which might be composed of clay minerals and CaCO_3_ from the carbonation of lime. An elastic modulus of 81.11 ± 15.66 GPa corresponds to the indentation on the silt and sand inclusions on a compliant substrate. A large array of indentations at other locations on the same TCC sample or on a different sample produces similar results, but with varying volume fractions (Table 2). This indicates a highly heterogeneous structure of the TCC at the mesoscale, as the indentation arrays for each trial of tests only cover a small area of the TCC surface, where different types of mechanical phases may exist in local zones.

**Figure 6:**
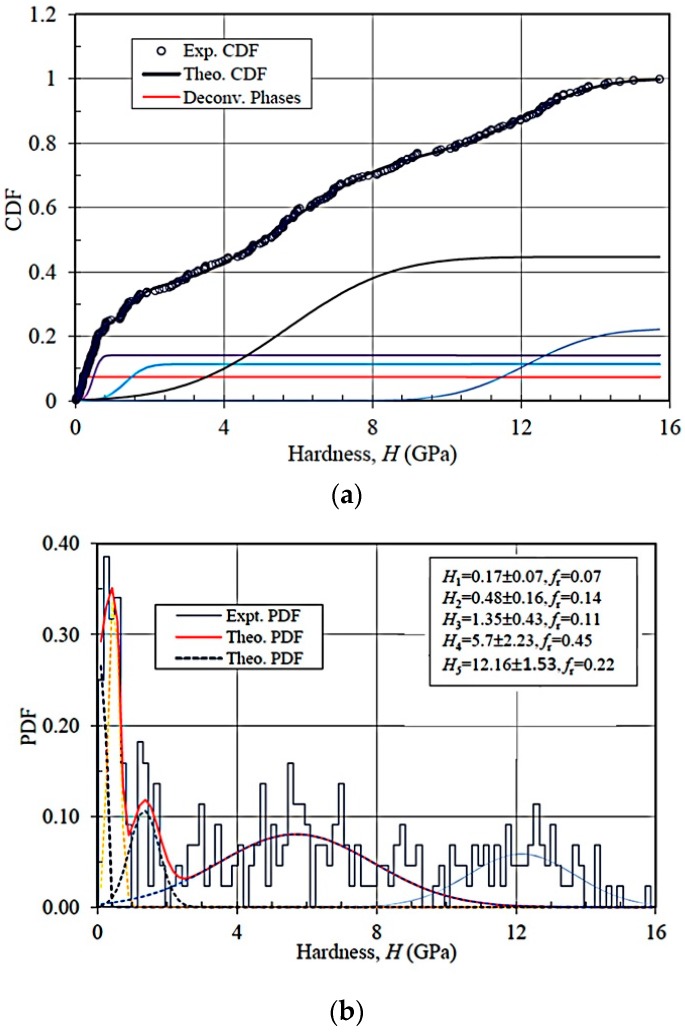
Reduced hardness deconvolution of ANC-1 data (**a**) CDF (**b**) PDF; *H*_i_: reduced modulus of phase *i* (*i* = 1: microporosity; *i* = 2: mixed geopolymer-clay matrix; *i* = 3: C-S-H; *i* = 4: nano-crystallites; *i* = 5: inclusions); *f*_r_: volume fraction.

Deconvoluted hardness data are illustrated in Figure 6 for trial ANC-1 and are summarized in Table 3 for all three indentation series. The lowest hardness value is the signature of indentation on the high porosity region. Hardness data for other peaks are consistent with the indentation moduli peaks shown in Figure 5 and Table 2. Like the modulus tests, the heterogeneous structure of the TCC at the mesoscale local zone leads to different volume fractions tested in the tests of the three trials.

### 3.4. Comparative Analysis

The hardened TCC material is composed of C-S-H gel, geopolymer gel, and crystalline CaCO_3_ as binder matrices, unreacted clay relics as filler, and sand and gravel fractions as aggregate, showing a highly heterogeneous microstructure. A semi-quantitative analysis on the TCC by XRD revealed five main minerals with their concentrations, as shown in Table 1, which correspond approximately to the five distinct mechanical phases. The peaks corresponding to the highest and lowest *E*_r_ (and *H*) show the indentation behavior in the presence of the microporosity and inclusion phases, respectively. A further three phases are identified as the signature of cementing binder phases.

#### 3.4.1. Quartz

The inclusion phase with the highest *E*_r_ and *H* corresponds to the quartz mineral contained in the raw material of TCC, sand. The *H* values of the inclusion phase are in good agreement with the reported value of 13.0 ± 0.7 GPa for quartz as reported by [33] in a nanoindentation test with *h*_p_ = 300~500 nm. However, the *E*_r_ value around 81.11 ± 15.66 GPa is slightly lower than the reported value of quartz 104.2 ± 5.9 GPa [33], which may be attributed to the porous structure of natural sand and the effect of surrounding materials on the micromechanical test of quartz particles in the TCC.

#### 3.4.2. C-S-H

The indentation modulus of 22.85~26.10 GPa is indicative of the C-S-H phase. Similar indentation tests performed on white cement or ordinary Portland cement (OPC) reported the indentation modulus in the range of 18.2 ± 4.2 to 21.7 ± 2.2 GPa for low density (LD) C-S-H and 29.1 ± 1 GPa to 31 ± 4 GPa for the high density (HD) C-S-H phases [34,35]. It is noted that the indentation modulus of C-S-H in OPC is highly dependent on the packing density and the water/cement ratio (w/c). The summary of the indentation modulus in Table 4 suggests that the C-S-H in the TCC is consistent with the LD C-S-H in OPC with a w/c ranging between 0.3~0.4 [21,22,23,24,25,26]. However, the *H* value for the C-S-H phase in TCC is higher than that for LD C-S-H in OPC, and is actually closer to that for HD C-S-H [26]. The ratio of *E*_r_ to $\sqrt{H}$ is related to the energy dissipation capacity of the material, which consists of the energy causing plastic deformation and the surface energy of microcracks generated during the loading/unloading process [36]. A lower dissipation capacity reveals a lower resistance to fracture in the C-S-H phase in the TCC materials than in the C-S-H phase in the OPC materials. This is likely to be caused by the difference between C-S-H, the pozzolanic reaction products between clay and lime in TCC, and the hydration products of Portland cement, in production process and, hence, in microstructures and micromechanical performance. This analysis needs to be further verified through experimental investigations.

#### 3.4.3. Calcite

In addition to a dispersive C-S-H skeleton, the presence of nanocrystallite with superior mechanical properties (*E*_r_ = 52.46~54.02 GPa, *H* = 5.57~5.70 GPa) is also identified in the TCC binder matrix. By simple estimation of the proportion by volume of each mineral, the nanocrystallite phase may be assigned to the feldspar and calcite minerals detected by XRD (Table 1). The indentation hardness of nanocrystallite agrees well with the reported value of 5.4 ± 1.0 GPa for albite, but the indentation modulus is slightly smaller than that of the albite with a value of 62.2 ± 6.0 GPa [33], which may have contributed to the lower packing density of natural albite particles existing in clay or sand in TCC. The indentation modulus of inorganic single crystal calcite was reported to be 73.5 ± 2.9 GPa [41] or 78.1 ± 5.2 GPa [42], which are much higher than that of the nanocrystallite in TCC. However, a lower indentation modulus of 59.1 ± 5.0 GPa was also observed in the outer part of a species of sea urchins with a porosity of 25% ± 2.8% [43], which suggests evidence for the presence of calcite in the TCC. In the carbonation process of lime with carbon dioxide, it is highly possible that a porous structure in the calcite similar to that found in the sea urchins may be formed, and this is likely to be due to the loose packing of calcium hydroxide and the adjustment effect of the sticky rice soup on the carbonation process of lime [6,10], which needs further investigations through reproduction of the complex chemical process.

#### 3.4.4. Geopolymer

The indentation modulus and hardness of the mixed clay-geopolymer matrix are in the range of 11.60 ± 5.4 GPa to 17.30 ± 2.58 GPa, and 0.48 ± 0.16 GPa to 0.62 ± 0.25 GPa, respectively, which are in good agreement with the reported values of 14 GPa and 0.5 GPa, in terms of modulus and hardness, respectively, for the geopolymer [44]. Additionally, the indentation on the sodium aluminosilicate hydrate by alkali-activated fly-ash shows an indentation modulus and hardness of 10.2 ± 4.54 GPa to 16.41 ± 5.67 GPa and 0.52 ± 0.19 GPa to 0.62 ± 0.27 GPa, respectively [45], which are consistent with the values of the mixed clay-geopolymer matrix in the TCC.

Based on the nanoindentation behavior, it can be inferred that the nano-mechanical behavior of the C-S-H and the geopolymer present in the TCC binder matrix are nearly identical to that produced in OPC concrete and alkali-activated fly-ash geopolymer materials. In particular, although the capacity for fracture resistance for the C-S-H in TCC seems to be slightly lower than that in OPC, the geopolymer and calcite, with similar content to C-S-H, enhanced the strength and durability of the TCC with the help of the sticky rice additive [6]. The coexistence and hybridization of multi-binders significantly enhancing the performance of TCC suggests a potential method to improve contemporary cement materials in terms of performance and durability.

## 4. Conclusions

The ternary clay-based composite widely used in historical construction and buildings in China consists mainly of C-S-H gel, geopolymer gel, or crystal CaCO_3_ as binders, unreacted clay relics as a filler, and sand as aggregate. Statistical deconvolution of a large volume of nanoindentation data suggests that the hardened TCC is composed of five major mechanically different phases. The nano-mechanical behavior of C-S-H and geopolymer present in the binder matrix of TCC are similar to that produced in Portland cement concrete and alkali-activated fly-ash geopolymer materials, while the calcite produced by the carbonation of lime in the TCC is close to the porous outer part of calcite in the shell of some sea urchins. Compared to OPC, the C-S-H contained in the TCC has a relatively lower ratio of indentation modulus to indentation hardness, implying a relatively lower resistance to fracture of the material. However, the geopolymer and calcite, at similar volumes to those in the C-S-H, help to enhance the strength and durability of the TCC by their higher energy resistance capacity or higher strength. Of course, the TCC material, which was usually hard to cast and slow to harden in practice, still needs great improvement in its workability and hardening rate if it is to be considered for cast-in-place constructions or pre-cast units of structures as a potential substitute of modern concrete.

## Figures and Tables

**Table 1:** Semi-quantitative analysis of TCC.

Mineral	Concentration (%)
Quartz	43.0
Feldspar	9.0
Gypsum/Anhydrite	2.0
Calcite	9.0
Amorphous Phase	37.0

**Table 2:** Reduced modulus of constituent phases obtained from statistical analysis of grid indentation results for three different trials.

Trials	Reduced Modulus	Amount of Data	Constituent Phase *i*				
			MP	MGC	C-S-H	NC	INC
ANC-1	*μ_i_*, GPa	300	5.82	12.94	22.85	54.02	81.11
	*σ_i_*, GPa		2.63	2.43	7.48	5.54	15.66
	*fr_i_*		0.08	0.09	0.19	0.18	0.46
ANC-2	*μ_i_*, GPa	100	8.18	17.30	26.10	52.46	79.40
	*σ_i_*, GPa		3.62	2.58	6.20	10.69	9.88
	*fr_i_*		0.31	0.26	0.19	0.21	0.06
ANC-3	*μ_i_*, GPa	100	4.80	11.60	24.30	53.40	84.00
	*σ_i_*, GPa		1.50	5.4	3.5	8.9	8.7
	*fr_i_*		0.19	0.43	0.17	0.19	0.02

MP: microporosity; MGC: mixed geopolymer-clay matrix; NC: nano-crystallites; INC: inclusions; *μ_i_*: mean value of modulus for phase *i*; *σ_i_*: deviation of modulus value for phase *i*; *fr_i_*: volume fraction of phase *i*.

**Table 3:** Hardness of constituent phases obtained from statistical analysis of grid indentation results for three different trials.

Trials	Hardness	Amount of Data	Constituent Phase *i*				
			MP	MGC	C-S-H	NC	INC
ANC-1	*μ_i_*, GPa	300	0.16	0.48	1.35	5.70	12.16
	*σ_i_*, GPa		0.07	0.16	0.43	2.23	1.53
	*fr_i_*		0.07	0.14	0.11	0.45	0.22
ANC-2	*μ_i_*, GPa	100	0.28	0.62	1.21	3.27	8.41
	*σ_i_*, GPa		0.10	0.25	0.11	1.94	0.45
	*fr_i_*		0.24	0.27	0.13	0.29	0.06
ANC-3	*μ_i_*, GPa	100	0.23	0.57	1.39	5.57	12.16
	*σ_i_*, GPa		0.15	0.19	0.55	2.20	1.53
	*fr_i_*		0.41	0.18	0.17	0.22	0.02

MP: microporosity; MGC: mixed geopolymer-clay matrix; NC: nano-crystallites; INC: inclusions; *μ_i_*: mean value of hardness for phase *i*; *σ_i_*: deviation of hardness value for phase *i*; *fr_i_*: volume fraction of phase *i*.

**Table 4:** Summary of nanoindentation properties of C-S-H.

Sample Info.	C-S-H	*E*_r_ (GPa)	*H* (GPa)	Method	Reference
w/c = 0.4	LD	21.7 ± 2.2	—	SNT	[37]
	HD	29.4 ± 2.4	—		
w/c = 0.35,	LD	23.4 ± 3.4	0.73 ± 0.15	SNT	[33]
	HD	31.4 ± 2.1	1.27 ± 0.18		
w/c = 0.5	LD	18.1 ± 4.0	—	SNT	[35]
	HD	31.0 ± 4.0	—		
w/c = 0.5, 5 months	LD	18.2 ± 4.2	0.45 ± 0.14	SNT	[34]
	HD	29.1 ± 4.0	0.83 ± 0.18		
w/c = 0.45	LS	22.89 ± 0.76	0.93 ± 0.11	SNT	[38]
	MS	31.16 ± 2.51	1.22 ± 0.07		
	HS	41.45 ± 1.75	1.43 ± 0.29		
w/c = 0.3	LD	23.7 ± 5.9	0.68 ± 0.18	SNT	[39]
	HD	36.1 ± 3.4	1.01 ± 0.16		
w/c = 0.2	LD	19.4 ± 4.8	0.44 ± 0.23	SNT	[40]
	HD	31.8 ± 6.1	0.88 ± 0.21		
w/c = 0.3	LD	21.9 ± 4.9	0.58 ± 0.12		
	HD	31.3 ± 4.5	0.87 ± 0.17		
w/c = 0.35	LD	25.6 ± 3.5	0.60 ± 0.10		
	HD	32.0 ± 2.9	0.87 ± 0.17		
w/c = 0.4	LD	22.5 ± 5.0	0.61 ± 0.17		
	HD	30.4 ± 2.9	0.92 ± 0.10		

LD: low density; HD: high density; LS: low stiffness; MS: medium stiffness; HS: high stiffness; SNT: statistical nanoindentation technique.
